# Visualization of ischemic stroke-related changes on ^18^F-THK-5351 positron emission tomography

**DOI:** 10.1186/s13550-018-0417-1

**Published:** 2018-07-16

**Authors:** Kuo-Lun Huang, Jung-Lung Hsu, Kun-Ju Lin, Chien-Hung Chang, Yi-Ming Wu, Ting-Yu Chang, Yeu-Jhy Chang, Chi-Hung Liu, Meng-Yang Ho, Shiaw-Pyng Wey, Tzu-Chen Yen, Nobuyuki Okamura, Ing-Tsung Hsiao, Tsong-Hai Lee

**Affiliations:** 1 Department of Neurology, Chang Gung Memorial Hospital, Taoyuan City, Taiwan; 2 College of Medicine, Chang Gung University, Taoyuan City, Taiwan; 3 Graduate Institute of Humanities in Medicine and Research Center for Brain and Consciousness, Taipei Medical University, Taipei, Taiwan; 4 Department of Nuclear Medicine and Center for Advanced Molecular Imaging and Translation, Chang Gung Memorial Hospital, Taoyuan City, Taiwan; 5 Healthy Aging Research Center and Department of Medical Imaging and Radiological Sciences, Chang Gung University, Taoyuan City, Taiwan; 6 Department of Radiology, Chang Gung Memorial Hospital, Taoyuan City, Taiwan; 7 Graduate Institute of Behavioral Sciences, Chang Gung University, Taoyuan City, Taiwan; 8 Division of Neuro-imaging, Institute of Development, Aging and Cancer, Tohoku University, Sendai, Japan; 9 Department of Pharmacology, Faculty of Medicine, Tohoku Medical and Pharmaceutical University, Sendai, Japan

**Keywords:** Ischemic stroke, ^18^F-THK-5351, Positron emission tomography, Diffusion tensor imaging, Astrogliosis, Neuroinflammation

## Abstract

**Background:**

The ^18^F-THK-5351 radiotracer has been used to detect the in vivo tau protein distribution in patients with tauopathy, such as Alzheimer’s disease and corticobasal syndrome. In addition, ^18^F-THK-5351 can also monitor neuroinflammatory process due to high affinity to astrogliosis. We aimed to explore ^18^F-THK-5351 distribution patterns and characteristics in patients with recent ischemic stroke.

**Results:**

Fifteen patients received ^18^F-THK-5351 positron emission tomography (PET) and diffusion tensor imaging (DTI) approximately 3 months after ischemic stroke. A region of interest (ROI) was placed in the peri-ischemic area and was mirrored on the contralateral side as the control, and a proportional value was derived from the ratio of the peri-ischemic ROI value over the mirrored ROI value. Increased ^18^F-THK-5351 retention was observed in the areas around and remote from the stroke location. The proportional ^18^F-THK-5351 values were negatively correlated with the proportional fractional anisotropy values (*r* = − 0.39, *P* = 0.04).

**Conclusion:**

^18^F-THK-5351 PET imaging provides a potential tool for in vivo visualization of the widespread ischemia-related changes associated with a microstructural disruption in recent ischemic stroke patients.

**Electronic supplementary material:**

The online version of this article (10.1186/s13550-018-0417-1) contains supplementary material, which is available to authorized users.

## Background

Tau protein plays an important role in microtubule assembly, and hyperphosphorylated tau (p-tau) protein may aggregate into neurofibrillary tangles as the hallmark of Alzheimer’s disease [1]. Total tau (t-tau) protein and p-tau protein accumulation have been interpreted as indicators of ongoing neuronal and axonal degeneration in Alzheimer’s disease [2]. ^18^F-THK-5351 is a recently developed radiotracer designed for the in vivo tau protein detection with PET imaging [3].

In addition to the affinity to tau protein, ^18^F-THK-5351 has also been reported to bind to monoamine oxidase B (MAO-B), which is attributed to its off-target binding in the striatum, thalamus, and brainstem [4, 5]. Furthermore, MAO-B is largely expressed in astrocytes, and reactive astrocytosis is a neuroinflammatory phenomenon in response to CNS acute injury or chronic neurodegeneration [6]. Over the recent decades, neuroinflammation has been considered as an essential contributor of CNS diseases, and that leads to increasing interest in visualizing neuroinflammatory changes in a non-invasive manner [7]. The binding affinity of ^18^F-THK-5351 to MAO-B has been applied to visualize neuroinflammation in vivo; in Ishibashi’s case report, ^18^F-THK-5351 PET imaging was done 2 years after an ischemic stroke, and it suggests increased ^18^F-THK-5351 retention along the pyramidal tract may represent Wallerian degeneration [8]. However, it is unknown how early ^18^F-THK-5351 retention can be observed after ischemic stroke. In the present study, we applied ^18^F-THK-5351 PET imaging and diffusion tensor imaging (DTI) to determine in vivo ^18^F-THK-5351 distribution patterns and associated microstructural characteristics in patients with recent ischemic stroke.

## Methods

### Participants

Patients with ischemic stroke were prospectively recruited from the Department of Neurology and Stroke Center at Linkou Chang Gung Memorial Hospital, Taiwan, for ^18^F-THK-5351 PET imaging from March 2016. Prior to enrollment, all participants were well informed of the study objectives and protocol and then provided their written informed consents. The study protocol and procedure for obtaining informed consent comply with the Helsinki Declaration and were approved by the institutional review board of Chang Gung Memorial Hospital (IRB No. 103-7584A) and the Governmental Ministry of Health and Welfare.

The inclusion criteria were as follows: (1) a diagnosis of acute ischemic stroke based on history, neurological examination, and brain MRI performed at the time of hospital admission and (2) no history of a previous disabling stroke. The exclusion criteria were as follows: (1) presence of a dementia diagnosis before the index stroke; (2) a mean score of the Chinese version of the Informant Questionnaire on Cognitive Decline in the Elderly ≥ 4; (3) a history of a substantial traumatic brain injury; (4) a history of epilepsy; and (5) cerebellar structural abnormality, such as old stroke and traumatic brain injury. The clinical stroke patterns were summarized by the Oxfordshire Community Stroke Project classification [9].

### MRI acquisition

Brain MRI was acquired at admission and 3 months after the stroke. The first MRI scan was acquired at admission mainly for ischemic stroke confirmation, and the scanning protocol included the fluid-attenuated inversion recovery (FLAIR), diffusion-weighted imaging (DWI), apparent diffusion coefficient (ADC), and T1W imaging sequences. Acute ischemic stroke was defined as lesions with hyperintensity on DWI and FLAIR images and hypointensity on ADC and T1W images.

Follow-up brain MRI scans were acquired around 3 months after the stroke onset. In addition to FLAIR and DWI sequences, the scanning protocol included an axial three-dimensional T1W MP-RAGE sequence and DTI sequence. The intervals from the index stroke to the MRI scanning were recorded in days. The parameters of each MRI sequence were summarized in Additional file 1.

### ^18^F-THK-5351 radiotracer preparation and PET imaging acquisition

The ^18^F-THK-5351 radiotracer was synthesized with a slight modification of the method by Harada et al. (details in Additional file 1) [3, 10]. Serial ^18^F-THK-5351 PET scans of the whole brain were acquired on a dedicated PET/CT scanner (Siemens Biograph mCT 16; Siemens Medical Solutions). Ten 1-min frames of PET images were acquired 50–60 min after an intravenous injection of 378 ± 17 MBq ^18^F-THK-5351 (^18^F-THK-5351 images) [11]. PET images were reconstructed using a three-dimensional OSEM (ordered subset expectation-maximization) algorithm (four iterations, 24 subsets; Gaussian filter = 2 mm; zoom = 3) with low-dose CT-based attenuation and scatter and random correction. The intervals from the index stroke to the ^18^F-THK-5351 PET study were recorded in days.

### Imaging processing and analysis

All the MRI and PET images were co-registered with T1W MP-RAGE images and resliced into 1 × 1 × 1 mm resolution by the SPM8. To obtain the standardized uptake value ratios (SUVRs) from ^18^F-THK-5351 PET images, the reference region was set at the individual’s cerebellar cortex. The DTI images obtained 3 months after the stroke were reconstructed using the ExploreDTI toolbox (http://www.exploredti.com) to generate the DTI parametric images including fractional anisotropy (FA), mean diffusivity (MD), axial diffusivity (AD), and radial diffusivity (RD).

### Region of interest analysis

To delineate the region of interest (ROI), ^18^F-THK-5351 PET SUVR images were overlaid on the co-registered T1W MP-RAGE images, and the DWI images from the acute stroke stage were used as the reference for the acute ischemic regions. An experienced neurologist who was blinded to patients’ clinical manifestations created a hand-drawn ROI. The ROIs were drawn on the peri-ischemic areas where substantial ^18^F-THK-5351 uptake was noted; the peri-ischemic area with the maximal diameter of ^18^F-THK-5351 uptake extent was selected as the target slice, and at least three consecutive slices were used for ROI creation. The ROI in the ipsilateral hemisphere was mirrored to the contralateral hemisphere [12]. Because of the heterogeneous nature of infarcts and the topographic distribution of ^18^F-THK-5351 uptake, the proportional values of the ^18^F-THK-5351 SUVRs and DTI parameters between bilateral ROIs were calculated (affected side divided by unaffected side) [13].

### Statistical analysis

Data were expressed as the median and interquartile range (IQR) or absolute number and proportion for descriptive statistics. The correlations among proportional values from ^18^F-THK-5351 SUVRs and DTI parameters were performed with non-parametric Kendall’s Tau-b correlation analysis. Statistical analyses were performed with SAS version 9.2 (SAS Institute Inc., Cary, NC, USA), and a *P* value < .05 was deemed significant.

## Results

Fifteen patients were enrolled; no recurrent stroke was noted during the follow-up period, and no adverse event after ^18^F-THK-5351 PET examination was reported. The demographic data, stroke clinical patterns, and vascular risk factors for each patient are summarized in Table 1. The median interval from the stroke occurrence to the DTI and ^18^F-THK-5351 scanning was 82 days (IQR, 63–92 days) and 102 days (IQR, 79–118 days), respectively.

**Table 1:** The demographic data and stroke clinical patterns for each patient

Case	Age	Sex	OCSP	MRS	Vascular territory	Vascular risk factors
1	65	F	TACS	3	MCA	HTN, dyslipidemia, DM, Af
2	76	M	TACS	4	MCA	Af
3	72	F	POCS	2	PCA	Dyslipidemia, DM
4	61	M	POCS	1	PCA	HTN, dyslipidemia
5	70	M	PACS	2	MCA	HTN, gout, Af
6	67	M	PACS	2	MCA	HTN, dyslipidemia
7	66	F	PACS	4	MCA	HTN, dyslipidemia, DM
8	59	M	PACS	1	MCA	HTN, dyslipidemia
9	78	F	PACS	2	MCA	HTN, DM
10	80	F	LACS	1	MCA	HTN, CAD
11	61	M	LACS	1	MCA	HTN, dyslipidemia, gout
12	67	F	LACS	3	MCA	HTN, dyslipidemia
13	67	M	LACS	1	MCA	HTN, dyslipidemia, DM, CAD
14	75	F	LACS	1	MCA	HTN, dyslipidemia
15	69	F	POCS	2	VBA	HTN, dyslipidemia, DM

*OCSP* Oxfordshire Community Stroke Project, *MRS* Modified Rankin Scale, *TACS* total anterior circulation syndrome, *PACS* partial anterior circulation syndrome, *LACS* lacunar syndrome, *POCS* posterior circulation syndrome, *MCA* middle cerebral artery, *PCA* posterior cerebral artery, *VBA* vertebrobasilar artery, *HTN* hypertension, *Af* atrial fibrillation, *DM* diabetes mellitus, *CAD* coronary artery disease

**Fig. 1:**
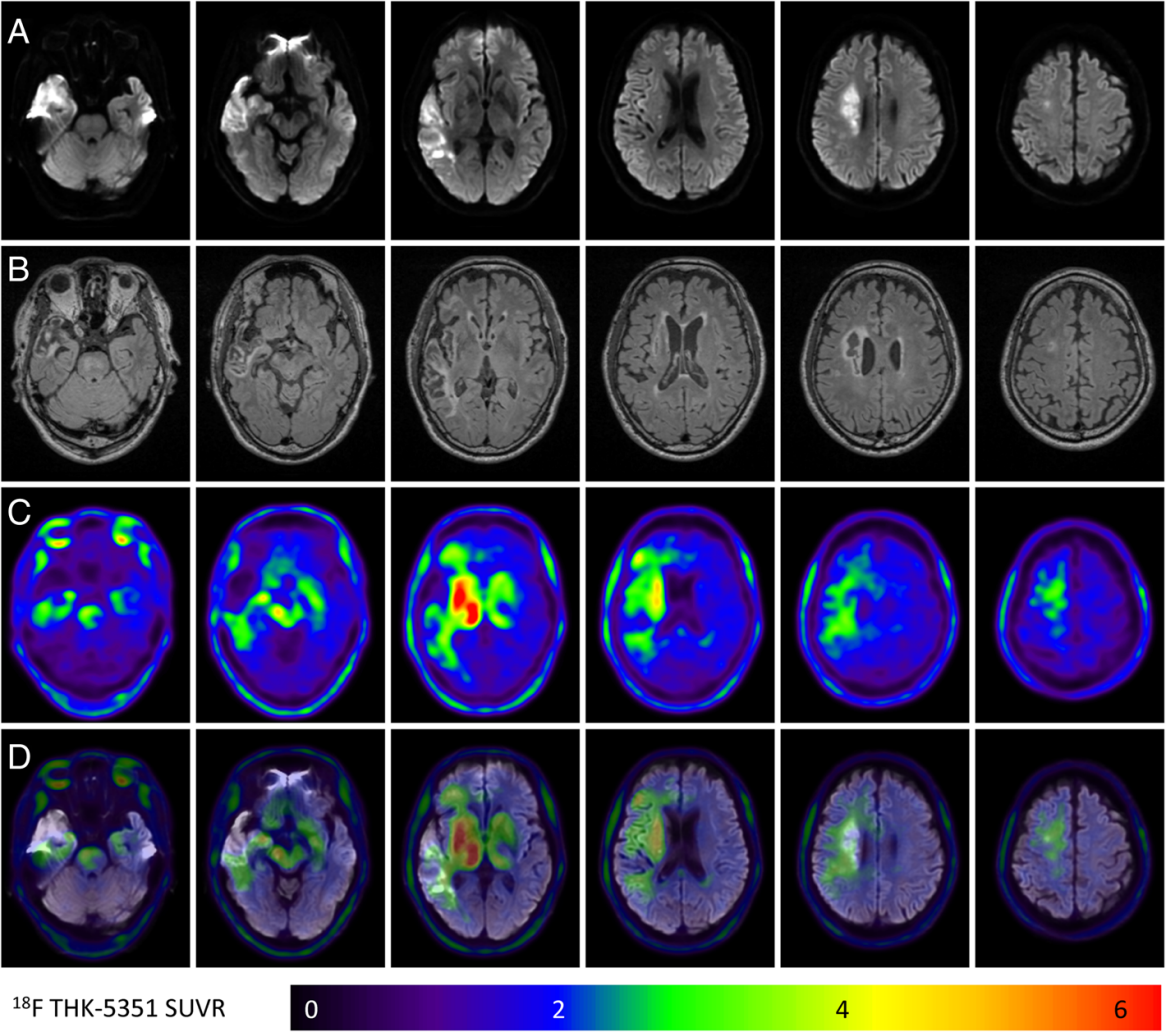
^18^F-THK-5351 retention patterns in an ischemic stroke patient (case 6). **a** The acute ischemic location on diffusion-weighted imaging (DWI). **b**, **c** The FLAIR images and ^18^F-THK-5351 PET images taken 3 months after stroke, respectively. **d** The fused DWI and ^18^F-THK-5351 PET images. Common off-target binding of the ^18^F-THK-5351 is observed in the bilateral striatum, thalamus, and brainstem. Additionally, the ^18^F-THK-5351 retention is asymmetrically increased in the remote and peri-infarct areas and decreased in the infarct core

Figure 1 shows representative images of a participant with an acute infarction in the right corona radiata and right temporal lobe (case 6). In addition to the common off-target binding of ^18^F-THK-5351 to the bilateral thalamus, striatum, and brainstem, more obvious ^18^F-THK-5351 retention is noted in peri-ischemic and remote areas over the right central semiovale, basal ganglion, right thalamus, right cerebral peduncle, and right ventral pons while low ^18^F-THK-5351 uptake is noted in the ischemic core.

**Fig. 2:**
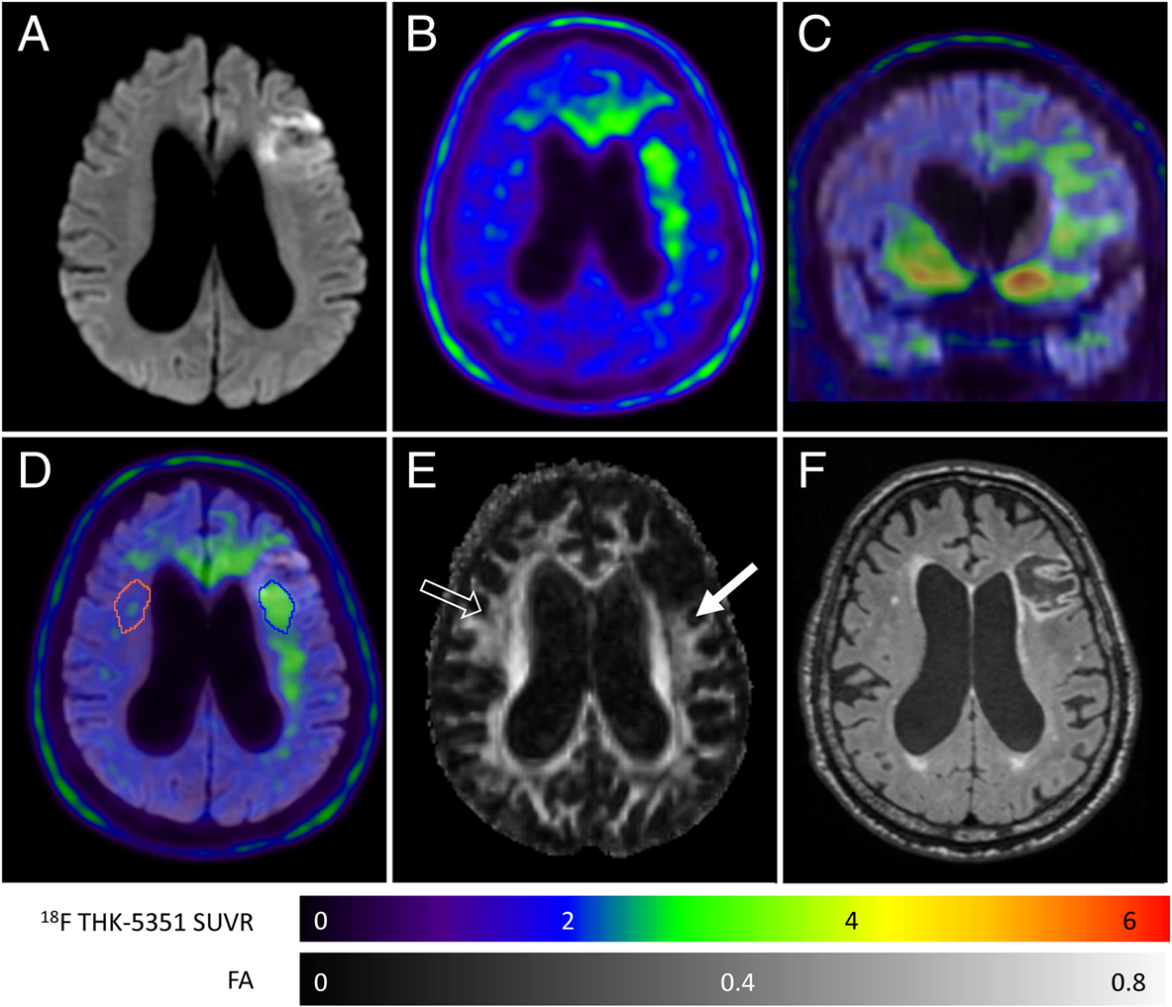
Multi-modality imaging characteristics in the patient (case 5) with a left frontal cortical infarction on diffusion-weighted imaging (DWI) (**a**). Low ^18^F-THK-5351 uptake is noted in the ischemic core, and high uptake is noted in the peri-ischemic area over the corona radiata, the genu of the corpus callosum, and in the bilateral frontal areas (**b**, **c**). The ROI for ^18^F-THK-5351 retention is drawn in the peri-ischemic area, and a mirrored ROI is placed in the contralateral hemisphere as the control (**d**). There is a decreased fractional anisotropy (FA) on diffusion tensor imaging (**e**) (filled arrow) in the peri-ischemic area with increased ^18^F-THK-5351 retention as compared to the contralateral side (empty arrow). No corresponding white matter change is noted on the follow-up FLAIR image (**f**)

Figure 2 shows representative images of another participant with an acute left frontal cortical infarction (Fig. 2a) (case 5). ^18^F-THK-5351 retention is decreased in the ischemic core and increased in the peri-ischemic areas (Fig. 2b, c) when overlaid on the DWI image (Fig. 2d). In the peri-ischemic areas with high ^18^F-THK-5351 retention, there is decreased FA on DTI (Fig. 2e) without corresponding white matter change on the follow-up FLAIR image (Fig. 2f).

**Fig. 3:**
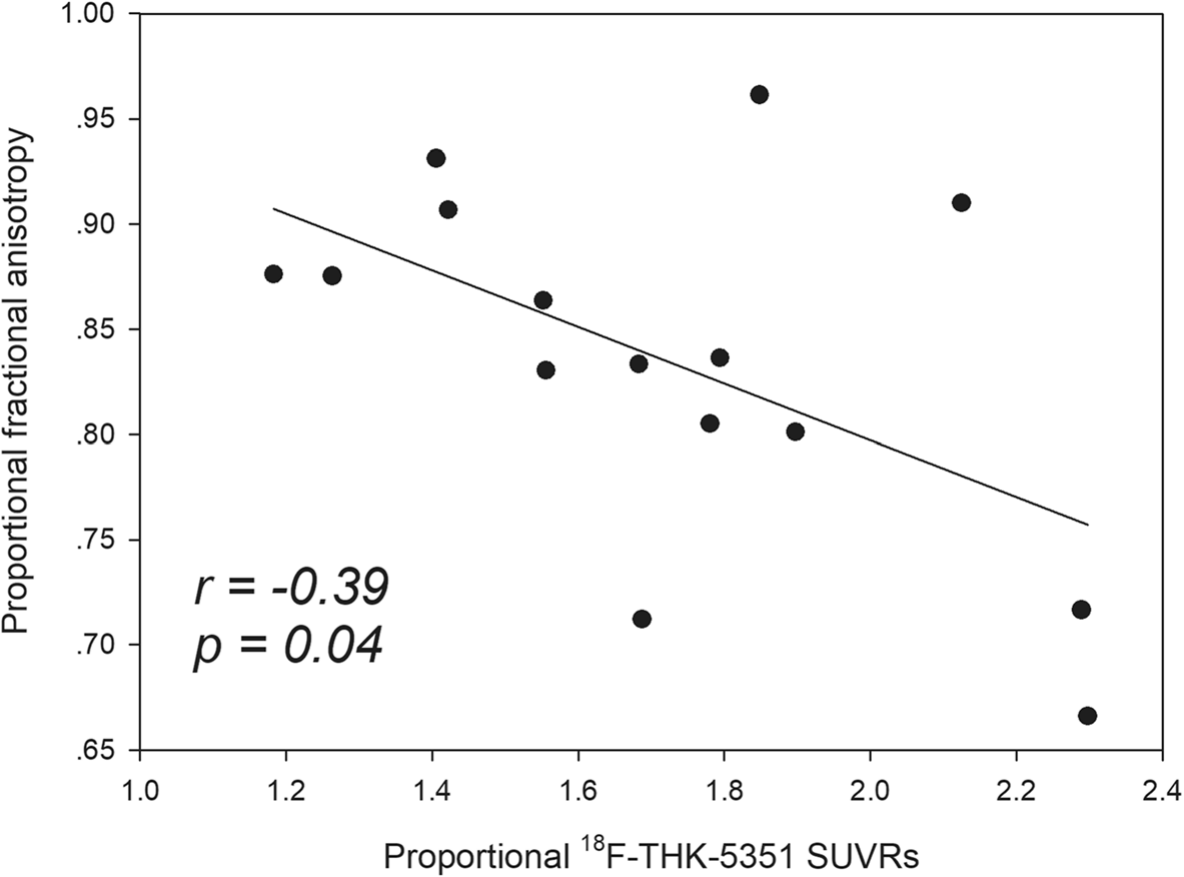
Correlation of the proportional ^18^F-THK-5351 standardized uptake value ratios (SUVRs) with the proportional fractional anisotropy (FA) values. The proportional ^18^F-THK-5351 SUVRs are negatively correlated with the proportional FA values, suggesting that peri-ischemic areas with higher ^18^F-THK-5351 retention have worse microstructural arrangements

The ROI data from the ^18^F-THK-5351 SUVRs and DTI parameters for each participant are listed in Additional file 1: Table S1. The proportional values of the ^18^F-THK-5351 SUVRs and DTI parameters are presented in Table 2. The proportional ^18^F-THK-5351 SUVRs are negatively correlated with the proportional FA values (*r* = − 0.39, *P* = 0.04) (Fig. 3), but not with other DTI parameters, such as proportional MD, RD, and AD.

**Table 2:** The proportional ^18^F-THK-5351 SUVR and diffusion tensor imaging parameters

Case	Proportional THK-5351 SUVR	Proportional fractional anisotropy	Proportional mean diffusivity	Proportional axial diffusivity	Proportional radial diffusivity
1	2.125	0.91	1.135	1.116	1.149
2	1.552	0.864	0.998	0.963	1.031
3	2.289	0.717	0.995	0.913	1.053
4	2.297	0.666	1.257	1.227	1.273
5	1.687	0.712	1.178	1.045	1.3
6	1.683	0.833	1.009	0.975	1.035
7	1.794	0.836	0.946	0.921	0.963
8	1.78	0.805	1.163	1.065	1.264
9	1.556	0.83	1.042	0.999	1.077
10	1.406	0.931	1.006	0.993	1.016
11	1.183	0.876	1.038	0.999	1.07
12	1.898	0.801	1.138	1.079	1.186
13	1.848	0.961	0.99	0.967	1.01
14	1.422	0.907	1.022	0.988	1.052
15	1.264	0.875	1.066	1.026	1.1

*SUVR* standardized uptake value ratio

## Discussion

Ischemic stroke has served as the prototypical models to investigate CNS neuroinflammatory response in both human and animal studies [6, 14]. The recently developed ^18^F-THK-5351 radiotracer has shown affinity to MAO-B, suggesting ^18^F-THK-5351 can demonstrate lesions with astrogliosis [5]. We conducted a case series study to explore the post-ischemic gliosis changes on ^18^F-THK-5351 PET imaging and DTI in patients with recent ischemic stroke. Firstly, increased ^18^F-THK-5351 uptake was mainly observed around the DWI-positive region as well as the remote areas. Furthermore, the proportional ^18^F-THK-5351 SUVRs were negatively correlated with the proportional FA values. To our knowledge, this is the first in vivo human case series study using ^18^F-THK-5351 PET scanning to determine ischemia-related changes in patients with a recent ischemic stroke event.

Despite the high affinity of ^18^F-THK-5351 for paired helical filaments, off-target binding of ^18^F-THK-5351 in middle-aged cognitively intact people was noted in the thalamus, striatum, substantia nigra, and periaqueductal gray matter where negligible tau protein was supposed to exist [15]. Similar off-target binding patterns were also reported with another tau PET tracer, ^18^F-AV-1451, and proposed to be associated with MAO and neuromelanin [16]. In a recent study by Ng et al., the off-target uptakes of ^18^F-THK-5351 in the thalamus and basal ganglia were reduced over 50% after administration of the MAO-B inhibitor selegiline [4]. In a recent postmortem Alzheimer’s disease study, regional in vivo ^18^F-THK-5351 uptake was significantly correlated with the density of tau aggregates in the neocortex and MAO-B in the whole brain [5]. Therefore, the findings with increased ^18^F-THK-5351 retention should be differentiated between tau protein accumulation and neuroinflammatory changes based on the underlying pathophysiology [6, 17–20].

A recent report has observed increased ^18^F-THK-5351 retention along the pyramidal tract in a chronic stroke patient [8], and elevated ^18^F-AV-1451 uptake was also noted in the peri-infarct areas with white matter changes [21]. Similar ^18^F-THK-5351 retention patterns in the peri-infarct areas were also noted in our patients with recent ischemic stroke. In fact, reactive astrocytosis is a phenomenon of morphological and functional changes to astrocytes in response to cerebral ischemia, and MAO is abundant in the outer membranes of astrocyte mitochondria [18, 22]. In a recent report by Thiel et al., the radiotracer, ^11^C-PK11195, was applied in patients with ischemic stroke, and antegrade increased retention was noted in the peri-lesional and remote areas along the pyramidal tract [13]. Such retention patterns of ^11^C-PK11195 may indicate post-ischemic astrogliosis [23], and their imaging presentations are quite similar to the ^18^F-THK-5351 uptake distribution in ischemic stroke patients. In fact, the well-known off-target binding sites of ^18^F-THK-5351, including the striatum, thalamus, and brainstem, were bilaterally enhanced in our cases, and the ^18^F-THK-5351 retention of these regions was asymmetrically elevated if they were within the peri-ischemic areas (Fig. 1). The superimposed ^18^F-THK-5351 imaging findings in ischemic stroke patients may suggest astrogliosis caused by different underlying mechanisms. Furthermore, the ^18^F-THK-5351 retention appeared more widespread as increased uptake was even noted in the subcortical areas and from the genu of the corpus callosum to the contralateral frontal areas (Fig. 2b, c). Such spillover imaging phenomenon may imply that the influence of ischemic injury is not limited to the infarct core and penumbra, and also, other brain regions where no macrostructural changes are observed on the MRI scans. Nevertheless, some possible off-target binding of ^18^F-THK-5351 cannot be excluded especially if the uptake is in the subcortical areas.

Although ischemic stroke was reported to induce tau protein accumulation in human postmortem and animal studies [19, 20], such tau protein accumulation may be a transient reaction. Tau protein was found to have the highest serum and cerebrospinal fluid concentration within 1 week after stroke onset [24, 25], and its level may gradually return to the baseline level 1 month later [26]. In our cases, the ^18^F-THK-5351 PET studies were performed about 3 months after stroke onset.

Diffusion tensor imaging was utilized to characterize ^18^F-THK-5351 uptake patterns in patients with recent ischemic stroke in our study. DTI has been applied to assess microstructural neuronal damage in ischemic stroke. In patients with a lacunar infarct, diffusion abnormalities are present in the affected tract up to 2 cm away from the lacune with decreased FA and increased diffusivity [27]. These findings are consistent with our study results showing that peri-ischemic lesions with increased proportional ^18^F-THK-5351 uptake had lower proportional FA, suggesting axonal degeneration or a demyelinating process in these areas.

There are several limitations to the current study. Firstly, the ^18^F-THK-5351 PET imaging and DTI were performed 3 months after the stroke event, and there may be dynamic changes of ^18^F-THK-5351 PET and DTI signals after the index stroke. Therefore, a longitudinal study investigating how ^18^F-THK-5351 retention evolves from the acute to the chronic ischemic stroke stage is warranted. Furthermore, there was heterogeneity in the stroke locations in our series. In order to determine the influence of ^18^F-THK-5351 retention patterns on stroke outcome, it is necessary to collect different groups of patients based on a pre-defined stroke location or syndrome in a larger sample size. Secondly, there has been a discussion about the tau radiotracer off-target binding characteristics, and the imaging findings of ^18^F-THK-5351 PET in stroke patients could be better elucidated if corresponding fluid biomarkers, tissue pathology, or other complement imaging results are available. Finally, further studies are required to evaluate the influence of increased ^18^F-THK-5351 uptake on long-term stroke prognosis and cognitive outcome.

## Conclusions

We have presented the first in vivo ^18^F-THK-5351 PET report in patients with recent ischemic stroke, in whom increased ^18^F-THK-5351 retention was noted in the areas around and remote from the stroke location. In lesions with increased ^18^F-THK-5351 retention, axonal degeneration or demyelination was found on DTI. Further studies are required to determine the influence of in vivo ischemia-related changes on ^18^F-THK-5351 PET on long-term stroke prognosis and cognitive outcome.

## Additional file

Additional file 1: **Table S1.** The standardized uptake value ratios (SUVRs) of ^18^F-THK-5351 PET and diffusion tensor imaging parameters in the bilateral cerebral hemispheres. (DOCX 45 kb)

## Abbreviations

AD: Axial diffusivity
ADC: Apparent diffusion coefficient
Af: Atrial fibrillation
CAD: Coronary artery disease
DM: Diabetes mellitus
DTI: Diffusion tensor imaging
DWI: Diffusion-weighted imaging
FA: Fractional anisotropy
HTN: Hypertension
IQR: Interquartile range
LACS: Lacunar syndrome
MAO-B: Monoamine oxidase B
MCA: Middle cerebral artery
MD: Mean diffusivity
MRS: Modified Rankin Scale
OCSP: Oxfordshire Community Stroke Project
OSEM: Ordered subset expectation-maximization
PACS: Partial anterior circulation syndrome
PCA: Posterior cerebral artery
PET: Positron emission tomography
POCS: Posterior circulation syndrome
p-tau: Hyperphosphorylated tau
RD: Radial diffusivity
ROI: Region of interest
SUVR: Standardized uptake value ratio
TACS: Total anterior circulation syndrome
t-tau: Total tau
VBA: Vertebrobasilar artery
