# Supplementary material for: Visualization of ischemic stroke-related changes on 18F-THK-5351 positron emission tomography
Source: EJNMMI Res. 2018 Jul 16;8:62. doi: 10.1186/s13550-018-0417-1 (PMC6047954; doi:10.1186/s13550-018-0417-1)
Supplement: Supplementary file 1 — Table S1. The standardized uptake value ratios (SUVRs) of 18F-THK-5351 PET and diffusion tensor imaging parameters in the bilateral cerebral hemispheres. (DOCX 45 kb) [file 13550_2018_417_MOESM1_ESM.docx]

Additional file

European Journal of Nuclear Medicine and Molecular Imaging Research

Online Supplement

Visualization of ischemic stroke-related changes on ^18^F-THK-5351 positron emission tomography

Kuo-Lun Huang^1,2†^, Jung-Lung Hsu^1-3†^, Kun-Ju Lin^4,5^, Chien-Hung Chang^1,2^, Yi-Ming Wu^6^, Ting-Yu Chang^1,2^, Yeu-Jhy Chang^1,2^, Chi-Hung Liu^1,2^, Meng-Yang Ho^7^, Shiaw-Pyng Wey^4,5^, Tzu-Chen Yen^4,5^, Nobuyuki Okamura^8,9^, Ing-Tsung Hsiao^4,5*^, and Tsong-Hai Lee^1,2*^

^1^ Department of Neurology, Chang Gung Memorial Hospital, Taoyuan, Taiwan

^2^ College of Medicine, Chang Gung University, Taiwan

^3^ Taipei Medical University, Graduate Institute of Humanities in Medicine and Research Center for Brain and Consciousness, Taipei, Taiwan

^4^ Department of Nuclear Medicine and Molecular Imaging Center, Chang Gung Memorial Hospital, Taoyuan, Taiwan

^5^ Healthy Aging Research Center and Department of Medical Imaging and Radiological Sciences, Chang Gung University, Taiwan

^6^ Department of Radiology, Chang Gung Memorial Hospital, Taoyuan, Taiwan

^7^ Graduate Institute of Behavioral Sciences, Chang Gung University, Taiwan

^8^ Division of Neuro-imaging, Institute of Development, Aging and Cancer, Tohoku University, Sendai, Japan

^9^ Department of Pharmacology, Faculty of Medicine, Tohoku Medical and Pharmaceutical University, Sendai, Japan

^†^These authors contributed equally to this work.

*Correspondence to: Tsong-Hai Lee, MD, PhD

Department of Neurology, Chang Gung Memorial Hospital

No.5, Fuxing St., Guishan, Taoyuan, Taiwan

Tel: +886 3 328 1200, Fax: +886 3 328 7226

E-mail: thlee@adm.cgmh.org.tw

*Correspondence to: Ing-Tsung Hsiao, PhD

Healthy Aging Research Center and Department of Medical Imaging and Radiological Sciences, Chang Gung University, Taiwan

E-mail: lp97ing@gmail.com

Supplementary Method

MRI acquisition

Brain MRI was acquired at admission and 3 months after the stroke. The first MRI scan was acquired at admission mainly for ischaemic stroke confirmation, and the scanning protocol included a FLAIR sequence (repetition time / echo time = 9,000/84 ms, slice thickness = 5 mm), DWI sequence (repetition time / echo time = 4,100/124 ms, slice thickness = 5 mm), ADC sequence (repetition time / echo time = 7,900/95 ms, slice thickness = 4 mm) and T1W imaging sequence (repetition time / echo time = 2,200/20 ms, slice thickness = 4 mm). Acute ischaemic stroke was defined as lesions with hyperintensity on DWI and FLAIR images and hypointensity on T1W images.

Follow-up brain MRI scans were acquired around 3 months after the stroke’s onset. In addition to FLAIR and DW sequences, the scanning protocol included an axial three-dimensional T1W MP-RAGE sequence (repetition time / echo time = 2000/2.67 ms, voxel size = 1 × 1 × 1 mm) and DTI sequence (repetition time / echo time = 8800/91 ms, b values = 0, 1000 s/mm^2^, 64 directions, voxel size = 2.2 × 2.2 × 2.2 mm).

Preparation of the ^18^F-THK-5351 radiotracer

Preparation of the ^18^F-THK-5351 radiotracer was described in our previous study [1]. The radiotracer was synthesized using the methods of Harada et al [2], with slight modifications. In brief, THK-5351 was prepared from its tosylate precursor (S)-2-(2-methylaminopyrid-5-yl)-6-[[2-(tetrahydro-2H-pyran-2-yloxy)-3-tosyloxy]propoxy] quinoline (THK-5352) according to the previously described method for synthesizing THK-5105 and THK-5117 [3]. ^18^F-THK-5351 was purified using semi-preparative high-performance liquid chromatography (column: Inertsil ODS-4; GL Sciences, Inc.; mobile phase: 20 mM NaH_2_PO_4_/acetonitrile (75:25), flow rate: 5.0 ml/min). ^18^F-THK-5351 was obtained at a radiochemical yield of 30 ± 4% (decay-corrected) with a radiochemical purity of > 95% and specific activity of 178.8 ± 32.1 TBq/mmol. The radiotracer was formulated in a saline solution containing ethanol (7%), sodium ascorbate (0.5%), and polysorbate 80 (0.15%) for clinical evaluation.

References

1. Hsiao I-T, Lin K-J, Huang K-L, Huang C-C, Chen H-S, Wey S-P, et al. Biodistribution and radiation dosimetry for the tau tracer 18F-THK-5351 in healthy human subjects. J Nucl Med. 2017;58:1498-503.

2. Harada R, Okamura N, Furumoto S, Furukawa K, Ishiki A, Tomita N, et al. 18F-THK5351: A novel PET radiotracer for imaging neurofibrillary pathology in Alzheimer disease. J Nucl Med. 2016;57:208-14.

3. Okamura N, Furumoto S, Harada R, Tago T, Yoshikawa T, Fodero-Tavoletti M, et al. Novel 18F-Labeled Arylquinoline Derivatives for Noninvasive Imaging of Tau Pathology in Alzheimer Disease. J Nucl Med. 2013;54:1420-7.

| **Additional file 1: Table S1.** The standardized uptake value ratios (SUVRs) of ^18^F-THK-5351 PET and diffusion tensor imaging parameters in bilateral cerebral hemispheres. | | | | | | | | | | | | | | | |
| --- | --- | --- | --- | --- | --- | --- | --- | --- | --- | --- | --- | --- | --- | --- | --- |
|  |  |  |  | ROI in the cerebral hemisphere ipsilateral to the stroke side | | | | |  | Mirror ROI in the cerebral hemisphere contralateral to the stroke side | | | | | |
| Case | Days to DTI | Days to PET | ROI voxel (mm^3^) | ^18^F-THK-5351 | FA | MD | AD | RD |  | ^18^F-THK-5351 | FA | MD | AD | RD |  |
| 1 | 188 | 107 | 10336 | 3.63 | 0.23 | 100.2 | 121.9 | 89.4 |  | 1.71 | 0.26 | 88.3 | 109.3 | 77.8 |  |
| 2 | 88 | 110 | 1283 | 2.36 | 0.36 | 81.3 | 114.9 | 64.5 |  | 1.52 | 0.42 | 81.5 | 119.4 | 62.6 |  |
| 3 | 58 | 136 | 592 | 3.77 | 0.16 | 83.2 | 94.7 | 77.4 |  | 1.65 | 0.22 | 83.6 | 103.8 | 73.5 |  |
| 4 | 78 | 102 | 1864 | 2.55 | 0.08 | 135 | 144.9 | 130 |  | 1.11 | 0.11 | 107.4 | 118.1 | 102.1 |  |
| 5 | 75 | 118 | 2684 | 2.56 | 0.28 | 100.6 | 127.8 | 87 |  | 1.52 | 0.4 | 85.4 | 122.3 | 66.9 |  |
| 6 | 91 | 147 | 4672 | 2.36 | 0.26 | 81.7 | 103.1 | 71 |  | 1.4 | 0.31 | 81 | 105.8 | 68.6 |  |
| 7 | 63 | 78 | 4816 | 2.96 | 0.21 | 87 | 105 | 78 |  | 1.65 | 0.25 | 92 | 114 | 81 |  |
| 8 | 116 | 165 | 1039 | 2.92 | 0.34 | 84 | 115.3 | 68.4 |  | 1.64 | 0.42 | 72.2 | 108.3 | 54.1 |  |
| 9 | 59 | 80 | 1108 | 2.76 | 0.26 | 87.6 | 111.4 | 75.7 |  | 1.78 | 0.31 | 84 | 111.5 | 70.3 |  |
| 10 | 103 | 118 | 1699 | 2.4 | 0.26 | 85.7 | 107.9 | 74.6 |  | 1.71 | 0.28 | 85.1 | 108.6 | 73.4 |  |
| 11 | 82 | 100 | 7443 | 1.9 | 0.3 | 84.8 | 112.6 | 71 |  | 1.61 | 0.35 | 81.8 | 112.7 | 66.3 |  |
| 12 | 64 | 93 | 3788 | 2.91 | 0.27 | 87.7 | 112.6 | 75.2 |  | 1.53 | 0.33 | 77 | 104.3 | 63.4 |  |
| 13 | 62 | 78 | 2526 | 3.17 | 0.32 | 73.3 | 97.7 | 61.1 |  | 1.72 | 0.34 | 74 | 101.1 | 60.5 |  |
| 14 | 86 | 73 | 3676 | 2.66 | 0.32 | 78 | 104.4 | 64.8 |  | 1.87 | 0.36 | 76.3 | 105.7 | 61.6 |  |
| 15 | 92 | 79 | 1964 | 1.87 | 0.28 | 75.4 | 98.2 | 64 |  | 1.48 | 0.32 | 70.7 | 95.7 | 58.2 |  |
| ROI: region of interest (ROI); FA: fractional anisotropy; MD, mean diffusivity; AD, axial diffusivity; RD, radial diffusivity. | | | | | | | | | | | | | | | |
